# A longitudinal study of justice characteristics among girls participating in a sex trafficking court program

**DOI:** 10.1186/s40352-020-00127-1

**Published:** 2021-01-06

**Authors:** Mekeila C. Cook, Ryan D. Talbert, Breanna Thomas

**Affiliations:** 1 Division of Public Health Practice, Meharry Medical College, 1005 Dr. DB Todd Blvd., Nashville, TN 37209 USA; 2 Department of Sociology, University of Connecticut , Storrs, CT USA; 3 Indiana University, Bloomington, IN USA

**Keywords:** Commercial sexual exploitation, Sex trafficking, Juvenile justice

## Abstract

**Background:**

Sex trafficking is a public health and social justice issue that has traditionally been addressed with criminal justice solutions. Because many sex trafficking survivors are incarcerated for crimes related to their exploitation, specialty, human trafficking courts were developed to offer resources and assistance to labor and sex trafficking survivors. This study assessed justice-involved youth participating in a specialty, anti-trafficking court program. The purpose of this study was to investigate justice-related outcomes of participants in a specialty court program. We examined: (1) the relationship between age at first citation and justice characteristics (number of bench warrants, number of citations, number placements, and number of times ran away); and (2) the number of months between first citation and enrollment into the program with the aforementioned justice characteristics. We used negative binomial models to estimate the relationships between age at first citation, number of months between first citation and program enrollment, with the four justice characteristics (*n* = 181).

**Results:**

Adjusted models showed that younger age at first citation was associated with significantly more bench warrants and citations while in the program. Likewise, fewer months between first citation and program entry was related to more bench warrants and citations.

**Conclusions:**

There is a need to evaluate the appropriateness of specialty, trafficking court programs in reducing continued justice involvement and these programs ability to meet the evolving needs of sex trafficking survivors over time. We recommend universal screening for trafficking indicators for all systems-involved youth and relocating trafficking specialty courts out of juvenile courts to dependency courts.

It is estimated that between 4500 to 21,000 adolescents and young adults are involved in the sex trade each year in the United States (Swaner, Labriola, Rempel, Walker, & Spadafore, 2016). Traditionally, these youth have been viewed as teen prostitutes and treated accordingly by law enforcement and juvenile courts. Being criminalized for their abuse, these youth are processed through the justice system for reasons directly connected to their sexual exploitation (e.g., prostitution) as well as in-directly for related-offenses such as running away.

Despite many efforts by child advocates to shift the response to sex trafficking from punitive to restorative, survivors of sex trafficking are still detained and incarcerated for offenses related to their exploitation. In response to the identification of trafficking survivors, the justice system seeks to address the illegal activities often associated with trafficking, while also providing services to survivors in an effort to reduce continued criminal behavior (Kendis, 2019; Musto, 2013). Thus, human trafficking courts were developed following the model of other specialty programs, like drug courts. These court programs were developed to assist survivors (Kendis, 2019), yet it is unclear whether or not delinquency behaviors desist as a result of participation in such court programs.

While utilizing specialty court programs is one approach to addressing commercial sexual exploitation of children (CSEC), since sex trafficked youth often come into contact with law enforcement, another approach is taking no intervention action on the part of law enforcement. Petrosino and colleagues suggests that youth exhibiting delinquent behaviors, may be best left alone, allowing for the behaviors to desist with time and maturity (Petrosino, Turpin-Petrosino, & Guckenburg, 2010). While this may be viable option for juvenile delinquency in general, the research is not conclusive on this approach for sex trafficked youth. Given, the multi-faceted nature of sex trafficking in which the roles of victim and perpetrator can become blurred (Myatt, 2019), leaving sex trafficked youth to desist in their sexual exploitation with no support may not be the best long-term solution. We lay the foundation for 1) why sex trafficked youth should be provided services to assist in their departure from ‘the life’ when they are ready and 2) using data, we provide a rationale for why the juvenile justice system may not be the most effect intervention approach for this vulnerable population.

We apply tenants of the Age-Graded Theory (Laub & Sampson, 1993) to examine whether age at entry into the juvenile justice system and time between initial justice involvement and enrollment into the specialty court program are associated with participants’ justice characteristics (i.e., citations, placements, bench warrants, and running away). Our research questions are: 1) what is the relationship between age at first citation and justice characteristics (i.e., citations, placements, running away and bench warrants)? 2) What is the association between number of months between first citation received and enrollment into the court program and justice characteristics (i.e., citations, placements, running away and bench warrants)? We test two hypotheses based on the likelihood of youths’ involvement with the criminal justice system. First, we expect that younger age at first citation is associated with more citations, placements, running away, and bench warrants. Second, we hypothesize that more time between the first citation and enrollment into the court program is associated with more citations, placements, running away and bench warrants.

## Background

### Defining sex trafficking in children

One of the challenges in understanding sex trafficking in children is the varied and overlapping definitions that originate from sources primarily focused on policy and legislation issues (Musto, 2013). Here we discuss two primary definitions, commercially sexual exploited children and domestic minor sex trafficking. Commercial sexual exploitation of children (CSEC) is the abuse of, and crimes against children for sexual purposes in which a transaction of money or other goods/services are exchanged (Clayton, Krugman, & Simon, 2013). Domestic minor sex trafficking refers to US citizens and permanent residents who are children trafficked for sex in the United States. (Smith, Healy-Vardaman, & Snow, 2009; Smith, Mastrean, & Vardaman, 2010). The international guidelines of the Palermo protocol state that the term ‘trafficking’ applies to all youth under the age of 18, without the burden to prove coercion (United Nations, 2000). The Trafficking Victims Protection Act of 2000, designed to provide protection to trafficking survivors, goes further and defines sex trafficking of a minor under the age of 18 as “severe trafficking” (United Stated Department of Justice, 2000). Due to the overlapping definitions, we use the terms sex trafficked youth/survivors to refer to youth impacted by CSEC and DMST.

### Characteristics of commercially sexually exploited children

The characteristics of sex trafficked youth have been detailed in several previous studies (Barnert et al., 2017; Clayton et al., 2013; Estes & Weiner, 2001; Franchino-Olsen, 2019; Greenbaum, 2014; Ijadi-Maghsoodi, Cook, Barnert, Gaboian, & Bath, 2016; Reid, 2012). As prior research indicates, there are numerous risk factors for becoming involved in commercial sexual exploitation; the age at which exploitation occurs plays a significant role and varies by risk factors. Studies have shown that some risk factors for early age onset are running away, homelessness, and lack of resources (Cobbina & Oselin, 2011; Martin, Hearst, & Widome, 2010; Murphy, 2017; Saewyc, MacKay, Anderson, & Drozda, 2008). Several studies report a history of abuse as a risk for early age involvement in commercial sexual exploitation (Cobbina & Oselin, 2011; Kramer & Berg, 2003; Loza et al., 2010; Roe-Sepowitz, 2012). Alternatively, lack of resources to provide for basic needs, unemployment, pregnancy at an early age, and having dependent children were more commonly associated with later age of entry into commercial sexual exploitation (Cobbina & Oselin, 2011; Loza et al., 2010).

Adverse childhood experiences are common among sex trafficked youth (Naramore, Bright, Epps, & Hardt, 2017). One study of trafficked youth participating in a specialty court reported that 92% of the sample had a child abuse report investigated and substantiated by child protective services (Cook, Barnert, Ijadi-Maghsoodi, Ports, & Bath, 2018). Several studies of justice-involved youth investigate differences in adverse childhood experiences between those who had been trafficked versus those who had not been trafficked and found that those who had been trafficked had higher odds of physical and sexual abuse, higher odds of emotional and physical neglect as well as family violence, compared to those with no trafficking history (Cole, Sprang, Lee, & Cohen, 2014; Reid, Baglivio, Piquero, Greenwald, & Epps, 2017; Wilson & Widom, 2010). Youth who experience abuse in their household may be more inclined to run away to flee the abuse (Cobbina & Oselin, 2011; Murphy, 2017). Because runaway youth often do not have the resources to care for themselves, they may be vulnerable to recruitment into trafficking or may rely on survival sex to meet basic necessities, such as food and shelter (Murphy, 2017), both of which are a form of commercial sexual exploitation on the part of the trafficker and purchaser of sex.

### CSEC and length of time trafficked

The length of time youth are trafficked is an understudied area of research. Drawing from criminal justice research, it is clear that although “delinquent” behaviors tend to desist with age, the longer youth are engaged in delinquent behaviors the more challenging it is to change trajectories (Miller, Malone, Dodge,, & Conduct Problems Prevention Research, 2010; Ouderkerk & Ruppucci, 2010). A national study of youth and young adults involved in commercial sex reported that approximately 11% remained in “the life” for less than a year, 65% were involved for 1 to 4 years, and 24% spent 5 years or more in “the life” (Swaner et al., 2016). Escaping the trafficking situations proves very challenging for survivors, especially in the absence of the support necessary to escape and remain out of “the life”. Aside from the exploitation itself, other indicators of continued exploitation include episodes of running away, arrests/delinquency and truancy (Cole et al., 2014; Greenbaum, 2014). In a study among adult sex trafficking survivors, the longer victims spent in the trafficked situation the more difficult it was for them to respond to services made available to them (Muftic & Finn, 2013).

### CSEC and justice involvement

Criminology research indicates that engagement in the juvenile justice system often leads to further delinquency and justice involvement (Ouderkerk & Ruppucci, 2010; Ryan, Williams, & Courtney, 2013). Relatedly, youth experience worse health and behavioral outcomes the longer they are involved in the juvenile justice system (Zajac, Sheidow, & Davis, 2015). In many states, sex trafficking survivors are repeatedly incarcerated for crimes related to their exploitation (Geist, 2012). Historically, the response to commercial sexual exploitation has been punitive, resulting in youth being cited and detained for offenses like prostitution (Musto, 2013; Swaner et al., 2016). From 2008 to 2018, approximately 260 youth were arrested for prostitution in the U.S., a gross under-representation of trafficking survivors (OJJDP, 2019). Youth caught in a commercial sex act may be cited for other offenses (Shared Hope International, 2018; Smith et al., 2009). For example, one-report states that only 16% of youth and young adults who report trading sex were arrested for prostitution, yet 65% of them reported being arrested for other citations (Swaner et al., 2016). Detainment for their exploitation or related offenses makes it challenging for sex trafficking survivors to disengage from the justice system without appropriate support.

### CSEC and safe harbor policies

Each state mandates a legal age in which a person can legally consent to sexual activity. These laws often conflict with law enforcement action to arrest youth (who legally cannot consent to sex) for prostitution. Safe Harbor policies were enacted to address this conflict in the law. Safe Harbor policies are state-level legislations that dissuade prosecution of children who are trafficked, provides victims protection from the exploiter(s), and seek to prosecute exploiters and abusers (Polaris Project, 2014; Geist, 2012; Polaris Project, 2015; Wasch, Schilling Wolfe, Levitan, & Finck, 2016). These policies vary by state and offer either decriminalization, diversion, or a combination of both (Polaris Project, 2014; Wasch et al., 2016; Williams, 2017).

Partly due to Safe Harbor policies, law enforcement and juvenile courts began shifting from a punitive response to a restorative approach to CSEC; thus, moving away from charging youth with prostitution (Shared Hope International, 2018; Swaner et al., 2016; Wasch et al., 2016). As of 2016, there are 51 anti-trafficking courts currently operating in 18 states (Global Health Justice, 2018). Each specialty court operates differently and varies in the application of Safe Harbor policies. These courts have come under scrutiny by advocates in recent years. Although seen as an alternative to detention or incarceration by the justice system, advocates argue that such courts may not be as effective as some claim and may in fact re-traumatize and stigmatize participants (Kendis, 2019; Musto, 2013). Despite the burgeoning efforts across the country to develop specialized courts to address commercial sexual exploitation, the lack of well-established intervention strategies reflects a poor understanding of the unique needs of youth who participate in these programs and how best to deliver services.

### Theoretical framework: age graded theory

The gendered and age-graded nature of sex trafficking presents a unique challenge to addressing the issue. Because sex trafficking recruitment is centered on traffickers exploiting the vulnerabilities of their victims, it is unsurprising that children, who are reliant upon legal guardians to provide their basic needs and females, who have less social capital than do males, are often targets of traffickers’ efforts (Reid, 2012). Thus, the intersection of gender and age make adolescent girls vulnerable to recruitment into sex trafficking. In an effort to contextualize the factors that contribute to justice related outcomes for trafficked girls participating in a specialty court program, we apply portions of the Age-Graded Theory of Informal Social Control developed by Sampson and Laub (Laub & Sampson, 1993). The theory postulates that instability and disruptions in social control processes, like family unity, hinders children’s ability to develop positive social bonds. These social control processes during childhood and adolescence influence the risk of involvement in juvenile delinquency (Laub & Sampson, 1993).

Chohaney (2016) expanded the application of Age-Graded Theory of Informal Social Control, highlighting the influence of informal social control processes on victimization during childhood and adolescence. Reid and Piquero (2014) and Chohaney (2016), stress the adverse role that caregiver problems, unstable upbringing, family violence, delinquent siblings and peers, and lack of school involvement may contribute to vulnerability to CSEC (Chohaney, 2016; Reid & Piquero, 2014).

Given the potential adverse effects and harmful consequences frequently associated with CSEC, some researchers emphasize identifying age-graded risks and life circumstances that make adolescents vulnerable to becoming involved in commercial sexual exploitation (Reid, 2012; Wilson & Widom, 2010). Risk factors have varying impact on entry into sex trafficking depending on age or developmental life stage. Age of entry, or age of delinquency onset has been a common focus in the criminal career literature (Farrington et al., 1990). To understand pathways into trafficking, Reid and Piquero established the importance of age of onset of offending (2014). Research investigating the correlates of onset as well as the link between age of onset/ entry and subsequent offending patterns shows that an early age of onset is predictive of a longer delinquency/criminal trajectory (Piquero, Farrington, & Blumstein, 2003).

While prior research sought to understand the age-graded predictors of entry into commercial sex trafficking, the current study fills a gap in the literature by not only seeking to further contextualize the role of age and delinquency but to also assess the age-graded predictors of desistance among girls with a history of sex trafficking victimization. Taking this approach to Age Graded Theory, we conceptualize the age in which trafficked youth receive their first citation as the start of their justice involvement and enrollment into the anti-trafficking program as the social capital impetus to desist from further justice involvement. We capture risk for continued justice involvement using the number of bench warrants received, number of run-away episodes, and number of new citations after enrolling in the anti-trafficking program. We expect to find that trafficked youth who enter the juvenile justice system at younger ages and who stay in the justice system longer without proper services to address their trauma will have worse justice-related outcomes over time.

### Data

We used secondary data from a specialty anti-trafficking court program for survivors of commercial sexual exploitation within a southern California juvenile delinquency court system. Case files consist of administrative data collected from multiple sources including: the juvenile court, the department of children services, and other youth serving agencies. The specialty court offers resources and services to address trauma associated with trafficking and seeks to prevent further justice involvement. Unlike standard juvenile courts, this specialty court encourages regular court appearances to ensure that the needs of the participant are being met by the appointed service agencies. Consistent court appearances are an indication of active program engagement. Additional details about the program and participants is published elsewhere (Cook et al., 2018).

Data for this study were extracted from juvenile case files for the program from 2012 to 2014 (*n* = 184) and include demographic characteristics, mental health status, alcohol and substance use, child protective services history, and citation and detention history. Descriptive, bivariate, and multivariate analyses were performed to examine four justice characteristics measures—bench warrants, citations, running away, and placements. All participants were cis-gendered female and 74% were African American and 96% were US citizens. One participant went 72 months between first citation and program entry; we deleted this observation due to its status as an outlier. Two participants were missing information on abuse history and were excluded from analyses; analyses utilize information from 181 participants.

## Methods

The dependent variables are counts of bench warrants, citations, placements, and running away issued while participants were in the court program. A bench warrant is a court order that directs youth’s detainment if they encounter law enforcement. For this specialty court, bench warrants were issued for running away and failure to appear for a scheduled court visit. Citations include any offense or infraction on the part of the youth recorded by law enforcement and presented to the juvenile court. Examples of citations include assault, truancy, curfew violation, or possession of drug paraphernalia. Placements are court ordered temporary relocations to housing outside of the legal guardian’s residential home (i.e., group homes or foster care). Finally, running away is as unapproved leave from the youth’s residential home or from the court ordered placement.

The key independent variables are age at first citation (i.e., entry into the juvenile justice system) and the number of months between first citation and entry into the court program. We calculated age at first citation by subtracting participants’ date of birth from the date of the first citation. We calculated months between first citation and court entry by subtracting the date of the first citation from the start date for the court program. We also included a measure for the length of time trafficking survivors participated in the court program—also captured in months—to reduce potential bias in estimating justice characteristics for participants that varied in time in the program. In order to increase confidence that relationships between key independent and dependent variables are not the result of prior risk behaviors, we incorporated a measure of participants’ justice characteristics prior to program entry corresponding to the dependent variable in each model (i.e., baseline bench warrants, baseline citations, baseline placements, and baseline running away). We also included a measure of abuse history that indicates whether participants experienced some form of abuse (e.g., physical, sexual, or general neglect) prior to entry into the program. No history of abuse is the reference category.

We first present a description of participants in the specialty court program and summarize key measures. We then present bivariate correlations between dependent variables and the two key independent variables. Our primary analyses are four negative binomial regression models that examine the relationships between age at first citation, months between first citation and entry into the court program, baseline justice characteristics, abuse history, and four dependent measures—counts of bench warrants (*mean* = 1.77; *variance* = 3.10), citations (*mean* = 1.73; *variance* = 1.80), placements (*mean* = 1.92; *variance* = 3.39), and number of times ran away (*mean* = 1.06; *variance* = 1.77). We utilized negative binomial models due to our four dependent variables being over dispersed count variables with no upper bound (Cameron & Trivedi, 1998; Hilbe, 2011; Long, 1997). To ensure our findings were not due simply to our modeling strategy, we also ran Poisson models and found that results were substantively identical to those presented. We present results in average marginal effects and incident rate ratios with robust standard errors. Average marginal effects (*AMEs*) provide a substantive and practical interpretation of findings. AMEs are the estimated differences after the other variables in the model have been controlled for (Long & Freese, 2014; Williams, 2012). Finally, we graphed the adjusted predictions at representative values for our two key independent measures.

We employed theoretically motivated models that balance our relatively small sample size and the social control processes in Age-Graded theory. The models include our two key predictor variables—age at first citation, and months between first citation and program entry—and control for number of months a person was in the program, whether the participant had a history of abuse, and baseline measures for the outcomes of interest respective to each model (i.e., number of bench warrants, citations, placements, and ran away). University Institutional Review Board approved this secondary data analysis from review.

## Results

Table 1 shows a description of participants in the specialty court program between 2012 and 2014. We assessed justice characteristics while participants were in the program (i.e., the key dependent variables) and before participants entered the anti-trafficking court program (i.e., baseline). While in the program, participants averaged 1.78 bench warrants (*SD* = 1.76), 1.75 citations (*SD* = 1.80), 1.94 placements (*SD* = 1.84), and ran away an average of 1.07 times (*SD* = 1.31). The average age at first citation was 15.18 years old (*SD* = 1.41), and approximately 15.26 months passed between receiving the first citation and entering the court program (*SD* = 14.09). Participants spent an average of 11 months in the program (*SD* = 7.38). Furthermore, there were some differences in justice characteristics prior to the youth entering the program. Participants received approximately 1.24 bench warrants (*SD* = 1.50), 3.20 citations (*SD* = 1.80), and 4.41 placements (*SD* = 4.41). Approximately 56% of participants ran away at least once prior to entering the program. Half of participants experienced some form of abuse. Ninety percent had abused substances, and 75% had at least one mental health condition.

**Table 1:** Description of participants in a specialty court program for commercially sexually exploited children, 2012–2014 (*n* = 181)

*Variables*	*Mean/Proportion*	*SD*	*Range*
*Justice characteristics* ^a^			
Bench warrants	1.78	1.76	0–7
Citations	1.75	1.80	0–8
Placements	1.94	1.84	0–8
Ran away	1.07	1.31	0–6
*Independent variables*			
Age at first citation (in years)	15.18	1.41	11–18
Months between first citation and court program	15.26	14.09	0–60
Months in the program	11.35	7.38	1–31
Bench warrants at baseline ^b^	1.24	1.50	0–5
Citations at baseline ^b^	3.20	2.09	1–10
Placements at baseline ^b^	4.41	4.97	0–20
Ran away at baseline ^b^	0.56	–	–
Abuse history (yes = 1)	0.50	–	–
History of substance use (yes = 1)	0.90	–	–
Mental health condition (yes = 1)	0.76	–	–
Black (yes = 1)	0.74	–	–
U.S. citizen (yes = 1)	0.96	–	–

*Note*. Means, standard deviations (*S.D*.), and ranges presented for continuous variables. Proportions presented for categorical variables

^a^Dependent variables are counts of events while participants were in the specialty court program

^b^Baseline variables are events prior to entry into the court program

A correlation matrix between key measures is presented in Table 2. All four justice characteristics measures were positively correlated. In other words, a participant that received a high number of bench warrants while in the program also tended to receive high numbers of citations, placements, and ran away more often. When assessing age and justice characteristics, we found that younger age at first citation was associated with more bench warrants, citations, placements, and running away. Similarly, there was a negative association between the number of months between first citation and program entry with justice characteristics. Correlations showed that fewer months between first citation and program entry was associated with more bench warrants, citations, and running away. Finally, at the bivariate level, months between first citation and program entry was negatively related to age at first citation.

**Table 2:** Correlations between justice characteristics from participants in a specialty court program for commercially sexually exploited children, 2012–2014 (*n* = 181)

		(1)	(2)	(3)	(4)	(5)	(6)
(1)	Bench warrants	1.00	–	–	–	–	–
(2)	Citations	.86^*^	1.00	–	–	–	–
(3)	Placements	.67^*^	.66^*^	1.00	–	–	–
(4)	Ran away	.74^*^	.61^*^	.81^*^	1.00	–	–
(5)	Age at first citation	−.24^*^	−.28^*^	−.26^*^	−.13^*^	1.00	–
(6)	Months between first citation and court program	−.19^*^	−.18^*^	−.12	−.16^*^	−.54^*^	1.00

*Note*.^*^p ≤ .05

Table 3 displays negative binomial models estimating justice characteristics. Results are presented in incident rate ratios (*IRR*) with robust standard errors (*SE*), and average marginal effects (*AME*). For ease of interpretation, we include average marginal effects, which are interpreted as the predicted increase in the outcome based on a unit increase in the independent measure while other factors are held constant. Additionally, for parsimony, we focus on results for our two key variables of interest. The first model estimated the number of bench warrants issued while in the program. Results showed that a year increase in age at first citation was associated with .252 fewer bench warrants while in the court program, all other factors being held constant (*IRR* = .867, *SE* = .050; *p* < .05). A month increase between one’s first citation and entering the program was associated with a decrease in the number of bench warrants received by .039 (*IRR* = .978, *SE* = .006; *p* < .01). In other words, younger age at first citation and shorter time between first citation and program entry were associated with more bench warrants issued.

**Table 3:** Negative binomial models estimating justice characteristics for participants in a specialty court program for commercially sexually exploited children, 2012–2014 (*n* = 181)

*Independent Variables*	Model 1			Model 2			Model 3			Model 4		
	Bench warrants			Citations			Placements			Ran away		
	*IRR*	*(SE)*	*AME*	*IRR*	*(SE)*	*AME*	*IRR*	*(SE)*	*AME*	*IRR*	*(SE)*	*AME*
Age at first citation (in years)	.867^*^	(.050)	−.252	.852^**^	(.050)	−.277	.898	(.053)	−.209	.918	(.077)	−.093
Months between first citation and court program	.978^**^	(.006)	−.039	.986^*^	(.006)	−.025	.991	(.006)	−.018	.982	(.009)	−.019
Months in the program	1.072^***^	(.010)	.122	1.082^***^	(.009)	.137	1.072^***^	(.010)	.135	1.073^***^	(.015)	.076
Abuse history (yes = 1)	1.352^**^	(.149)	.533	1.286^*^	(.133)	.436	1.360^**^	(.158)	.598	1.564^*^	(.288)	.483
Bench warrants at baseline ^a^	1.114^*^	(.052)	.191	–		–	–	–	–	–	–	–
Citations at baseline ^a^	–	–	–	1.008	(.023)	.014	–	–	–	–	–	–
Placements at baseline ^a^	–	–	–	–	–	–	1.018	(.011)	.035	–	–	–
Ran away at baseline ^a^	–	–	–	–	–	–	–	–	–	1.685^**^	(.322)	.564
Intercept	5.900	(5.894)	–	6.587	(6.720)	–	3.349	(3.553)	–	1.089	(1.599)	–
Pseudo R^2^	.171			.204			.160			.122		

*Note*. Incident rate ratios (*IRR*), robust standard errors in parentheses (*SE*), and average marginal effects (*AME*) presented

^a^Models control for justice characteristics prior to entry into the program corresponding to the outcome measures

^*^p < .05. ^**^p < .01. ^***^p < .001 (two-tailed tests)

Model 2 estimated the number of citations received while in the program. A year increase in age at first citation was associated with approximately .277 fewer citations while in the court program, controlling for covariates (*IRR* = .852, *SE* = .050; *p* < .01). Consistent with Model 1, younger participants tended to have worse justice outcomes while in the program. A month increase in the time between first citation and program entry was associated with a .025 decrease in the number of citations one received (*IRR* = .986, *SE* = .006; *p* < .05). The third model presents placements while in the court program. The statistically significant bivariate association shown in Table 2 between age at first citation and placements goes away in Table 3 after controlling for other measures. Similarly, months between first citation and program entry and was not associated with placements. Model 4 estimated the number of times a participant ran away while in the program. Age was not significantly associated with running away after including covariates, and months between first citation and program entry was not significant at the *p* < 0.05 level.

**Fig. 1:**
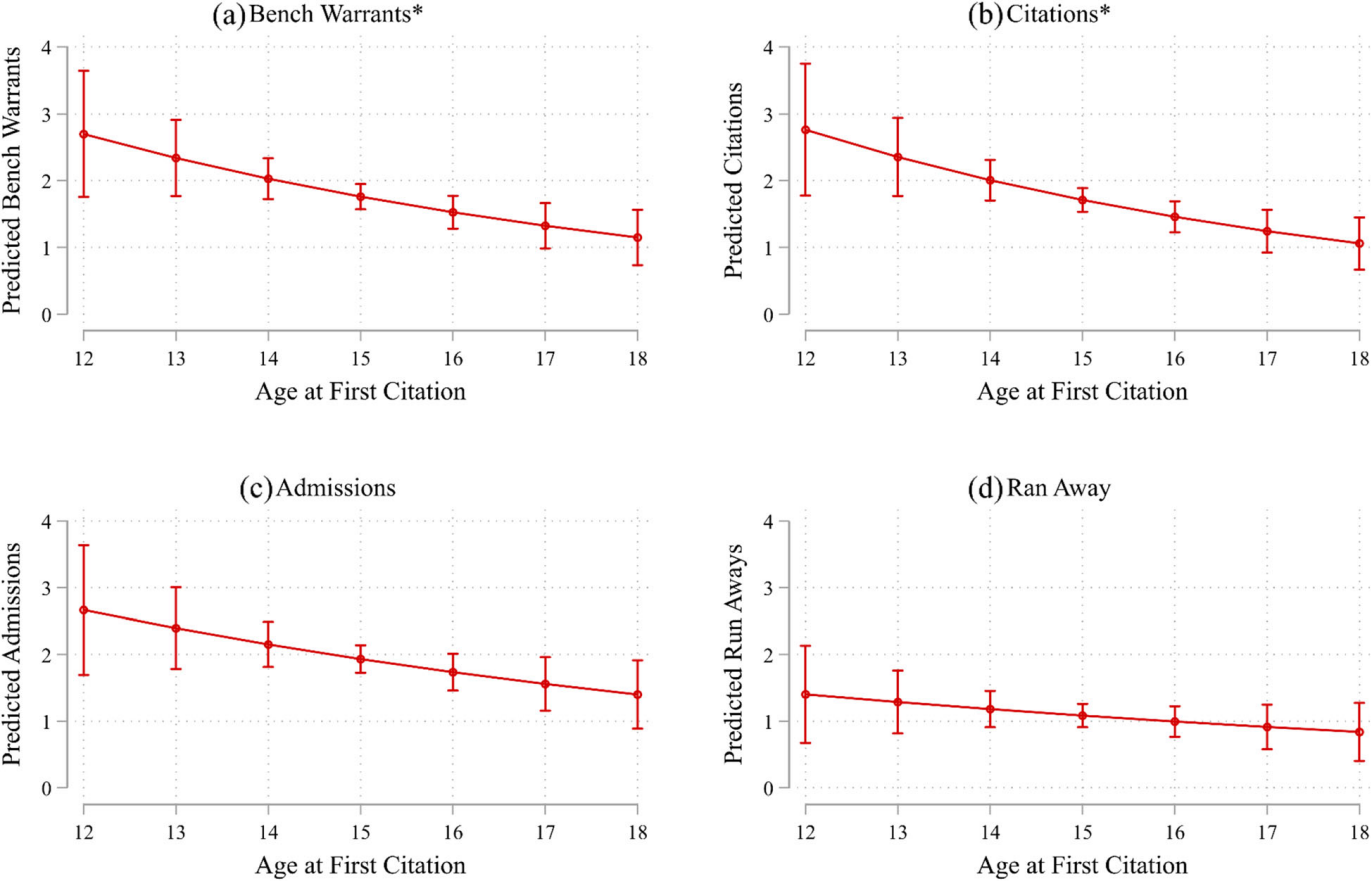
Predictions of Juvenile Characteristics by Age at First Citation for Participants in a Specialty Court Program for Commercially Sexually Exploited Children, 2012–2014 (*n* = 181). *Note*. Predictions estimated from models presented in Table 3. Asterisks denote significant decreases in juvenile characteristics

**Fig. 2:**
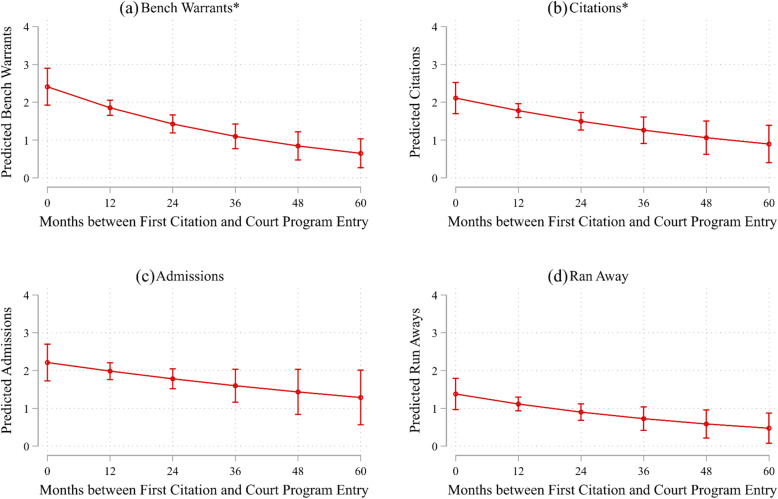
Predictions of Juvenile Characteristics by the Number of Months between First Citation and Program Entry for Participants in a Specialty Court Program for Commercially Sexually Exploited Children, 2012–2014 (*n* = 181). *Note*. Predictions estimated from models presented in Table 3. Asterisks denote significant decreases in juvenile characteristics

Figure 1 shows adjusted predictions of justice characteristics across the range of ages participants entered the specialty court program. Estimates for these graphs derive from models presented in Table 3. Figure 1a and b show significant declines in predicted justice characteristics outcomes as age at first citation increases. At age 12, a participant is predicted to receive 2.75 bench warrants and citations while in the program. By contrast, at age 18, participants would be expected to receive approximately one bench warrant and citation. The patterns are similar though not significant for number of admissions and number of runaway episodes. Moreover, Fig. 2 shows adjusted predictions of justice characteristics by the number of months between first citation and program entry. Across the outcomes, fewer number of months between first citation and program entry was associated with worse justice characteristics. Figure 2a and b show significant decreases in the number of bench warrants and citations as months between first citation and program entry increases. In summary, findings showed that younger age and less time between first citation and entry into the program were generally associated with worse justice characteristics.

## Discussion

The appropriateness of human trafficking court programs has been debated (Kendis, 2019; Musto, 2013). Though evaluating the effectiveness of the court program is beyond the scope of the present study, we addressed a gap in the literature by investigating the relationships between age at entry into the juvenile justice system, time between first citation and entering an anti-trafficking court program, and justice characteristics. We expected that younger age at entry into the justice system (i.e., age at first citation) would be associated with worse justice characteristics. Our hypothesis was supported. Younger age at entry into the juvenile justice system was associated with more (a) bench warrants and (b) citations. These findings align with prior research connecting abuse history and juvenile justice involvement. Saar and colleagues highlight that prior abuse is a pipeline to prison for adolescent girls. They further report that abuse that occurs at earlier ages and that last longer are predictors of delinquency in young girls (Saar, Epstein, Rosenthal, and Vafa (2015). Additional research using a mixed methods approach is necessary to contextualize the role of age at entry in the justice system and justice characteristics among sex trafficked youth. These findings point to a need for universal screening for trafficking indicators.

Second, we hypothesized that more time between first citation received and entry into the court program was associated with worse justice characteristics; our hypothesis was not supported. Shorter time between entry into juvenile justice system and entry into the court program was associated with worse justice characteristics. Trafficking survivors that go a longer period before encountering law enforcement may be more embedded in the trafficking world and know how to avoid detection. Consequently, their justice characteristics may generally appear better. On the contrary, we cannot say with certainty that justice characteristics are a holistic assessment of how at risk trafficked girls are. Other factors, such as homelessness, should be taken into consideration when assessing level of risk among sex trafficked girls. Alternatively, this finding lends support to investigate behavioral outcomes of youth who live in states where Safe Harbor policies have successfully decriminalized prostitution for minors and no legal intervention occurs. A study comparing behavioral and health outcomes in states with decriminalization policies versus states with diversion policies is warranted.

### Limitations

Although the present study has offered several contributions to the research literature, there are some limitations worth noting. The data used in this study come from a novel specialty court specifically designed for youth identified as trafficking survivors. As such, this sample is not representative of youth in the juvenile justice system or trafficking survivors overall. Ideal data would include a representative sample of trafficking survivors. Nevertheless, the data used here provide a unique compilation of information on a hard-to-reach population before participants entered the specialty court program and while they were in it. The data are best suited for the research questions addressed in this study, but do not allow us to disentangle the efficacy of the court program itself. Future research should investigate the role of specialty court programs in justice characteristics of trafficked youth. Additionally, the data used in this study do not assess the magnitude of survivors’ justice characteristics compared to other justice-involved girls with no sex trafficking history. Finally, we use legal terminology that may not align with some social justice and anti-trafficking movements. We acknowledge that this may be considered a limitation of the study. The findings presented in this study should be viewed considering the limitations stated above.

### Practice implications and future research

This study is one of the first to assess justice characteristics of sex trafficking survivors participating in a specialty court program. Findings from this study highlight the necessity for creating non-justice related interventions for survivors to prevent justice involvement altogether. We recommend employing community-based organizations to offer trauma-informed, wrap-around services to sex trafficked youth apart from the juvenile justice system. This will require a shift in policy and funding to better incorporate well-equipped community-based organizations as alternatives to justice related responses to CSEC. Our study illuminates the need to evaluate behavioral and justice-related outcomes from specialty courts to identify and test best practices to addressing CSEC in states that uphold diversion programs as an alternative to detention. This study further highlights the need to identify gaps in knowledge and opportunities for growth for similar court programs. We illustrate the importance of identifying survivors early in the trafficking process. Our findings reveal that younger girls are particularly vulnerable to worse justice characteristics, placing them at risk for continued exploitation and justice involvement. Thus, we recommend universal screening for trafficking indicators for all systems-involved youth. Screening will allow for prevention among vulnerable youth who have not been exploited, early detection of sex trafficking and more immediate non-justice related intervention for youth currently being sexually exploited.

This study contributes to the dialogue regarding sex trafficking interventions. Despite the support for human trafficking court programs as an alternative to detention, we recommend relocating these types of specialty courts out of the justice system altogether into dependency and/or family courts. Because dependency and family courts already offer social services to youth and their family, they may be better suited to provide comprehensive and trauma informed services. Movement out of the justice system aligns with the collective effort to address CSEC from a restorative approach instead of a punitive response. Additionally, dependency courts will allow a broader youth base to receive specific anti-trafficking services, removes the stipulation of committing a crime to receive services, is trauma-informed and reduces the stigma associated with being in the juvenile justice system.

Relatedly, future research should include information on samples of youth whereby comparisons can be made between trafficked youth and their non-trafficked counterparts. This future research should include youth from diverse race/ethnic backgrounds and explore gender differences in behavioral and justice characteristics. As we have mention moving the specialty court out of the justice system, future research should include an evaluation of a specialty court through the justice system versus an intervention that is not connected to the justice system.

## Conclusion

This study contributes to the larger public health and criminal justice literature by empirically addressing an important and immediate public health and social justice concern. We provide information on a hidden population and identify justice-related risk behaviors that can be used as an intervention point with trafficking survivors. While the purpose of this study is not to provide a determination whether or not the approach to sex trafficking should be “anti-prostitution” or “pro-sex work”, we would be remiss to forgo situating our findings without this conversation in mind. The purpose of this study was to meet a gap in the literature by assessing one approach (specialty court programs) for addressing commercial sexual exploitation of children. As reported, our findings indicate a need to move services designed to assist sex trafficking survivors out of the legal sector and lends support for decriminalization of prostitution among juveniles.

## Data Availability

The data that support the findings of this study may be available on reasonable request from the corresponding author, MC. The data are not publicly available due to their containing sensitive information that could compromise the privacy of participants.

## Appendix

**Table 4:** Poisson models estimating justice characteristics for participants in a specialty court program for commercially sexually exploited children, 2012–2014 (*n* = 181)

*Independent Variables*	Model 1			Model 2			Model 3			Model 4		
	Bench warrants			Citations			Placements			Ran away		
	*Logit*	*IRR*	*AME*	*Logit*	*IRR*	*AME*	*Logit*	*IRR*	*AME*	*Logit*	*IRR*	*AME*
Age at first citation (in years)	−.143^*^ (.058)	.867	−.252	−.160^**^ (.059)	.852	−.277	−.107 (.059)	.898	−.209	−.079 (.081)	.924	−.084
Months between first citation and court program	−.022^**^ (.006)	.978	−.039	−.014^*^ (.006)	.988	−.025	−.009 (.006)	.991	−.018	−.017 (.009)	.983	−.018
Months in the program	.069^***^ (.009)	1.072	.122	.079^***^ (.008)	1.082	.137	.069^***^ (.009)	1.072	.135	.064^***^ (.013)	1.066	.068
Abuse history (yes = 1)	.301^**^ (.110)	1.351	.531	.252^*^ (.104)	1.287	.436	.307^**^ (.116)	1.360	.598	.416^**^ (.184)	1.516	.441
Bench warrants at baseline ^a^	.108^*^ (.047)	1.114	.191	–	–	–	–	–	–	–	–	–
Citations at baseline ^a^	–	–	–	.008 (.023)	1.008	.014	–	–	–	–	–	–
Placements at baseline ^a^	–	–	–	–	–	–	.018 (.011)	1.018	.035	–	–	–
Ran away at baseline ^a^	–	–	–	–	–	–	–	–	–	.467^*^(.197)	1.595	.495
Intercept	1.780 (.992)	–	–	1.885 (1.020)	–	–	1.209 (1.061)	–	–	.113 (1.416)	–	–
Pseudo R^2^	.215			.255			.204			.156		

*Note*. Logits, incident rate ratios (*IRR*), and average marginal effects (AME) presented. Robust standard errors in parentheses

^a^Models control for justice characteristics prior to entry into the program corresponding to the outcome measures

^*^*p* < .05. ^**^*p* < .01. ^***^*p* < .001 (two-tailed tests)
