# Correction to: A longitudinal study of justice characteristics among girls participating in a sex trafficking court program

**DOI:** 10.1186/s40352-022-00180-y

**Published:** 2022-05-10

**Authors:** Mekeila C. Cook, Ryan D. Talbert, Breanna Thomas

**Affiliations:** 1 Division of Public Health Practice, Meharry Medical College, 1005 Dr. DB Todd Blvd, Nashville, TN 37209 USA; 2 Department of Sociology, University of Connecticut, Storrs, CT USA; 3 Indiana University, Bloomington, IN USA

**Correction to: Health Justice 9, 1 (2021)**

**https://doi.org/10.1186/s40352-020-00127-1**

Following the publication of the original article (Cook et al., 2021) the authors reported that URL of the reference “Glosser, Gardiner, & Fishman (2004)” is broken.

The reference with correct URL should be:

Glosser, A., Gardiner, K., & Fishman, M. (2004). Statutory rape: a guide to state laws and reporting requirements. Office of the Assistant Secretary for Planning and Evaluation https://aspe.hhs.gov/reports/statutory-rape-guide-state-laws-reporting-requirements-1

The original article (Cook et al., 2021) has been updated.

## References

[CR1] Cook M, Talbert R, Thomas B (2021). A longitudinal study of justice characteristics among girls participating in a sex trafficking court program. Health Justice.

